# The Association Between Handgrip Strength and Predialysis Serum Sodium Level in Patients With Chronic Kidney Disease Stage 5D

**DOI:** 10.3389/fmed.2020.610659

**Published:** 2021-01-12

**Authors:** Anastasia Markaki, Periklis Kyriazis, Eleftheria-Kleio Dermitzaki, Sevasti Maragou, Emmanuel Psylinakis, Aspasia Spyridaki, Helen Drosataki, Dimitra Lygerou, Maria G. Grammatikopoulou, Ioannis Petrakis, Kostas Stylianou

**Affiliations:** ^1^Department of Nutrition and Dietetics Sciences, Hellenic Mediterranean University, Crete, Greece; ^2^Division of Nephrology, Beth Israel Deaconess Medical Center, Harvard Medical School, Boston, MA, United States; ^3^Department of Nephrology, Heraklion University Hospital, Crete, Greece; ^4^Department of Nutritional Sciences and Dietetics, Faculty of Health Sciences, International Hellenic University, Thessaloniki, Greece; ^5^Department of Nephrology, Saarland University Medical Center, Homburg, Germany

**Keywords:** handgrip strength, hyponatremia, dialysis, nutrition, sarcopenia

## Abstract

**Purpose:** Handgrip strength (HGS) is a useful tool for the systematic assessment of muscle function related to nutritional status. Reduced HGS has been associated with adverse clinical outcomes in chronic kidney disease (CKD) stage 5D patients. In the same patients, predialysis low serum sodium (sNa) has been associated with malnutrition and mortality. Here, we investigated the role of predialysis sNa on muscle function in CKD-5D patients.

**Methods:** We evaluated 45 patients on hemodialysis (HD) and 28 patients on peritoneal dialysis (PD) with HGS measurement, bioimpedance analysis, anthropometric measures, and malnutrition inflammation score (MIS). According to established diagnostic criteria, reduced HGS was defined as strength below 30 and 20 Kg in men and women, respectively. Predialysis sNa values were defined as the mean of all predialysis measurements during the preceding 6 months. Data analysis was performed separately for each of the HD and PD groups.

**Results:** The proportions of reduced HGS did not differ between the HD (66%) and PD (54%) groups, respectively. Patients in the HD group as compared to those in the PD group had higher serum albumin and potassium and mid-arm muscle circumference and lower residual renal function (RRF) and residual urine volume. Multivariate logistic analysis, after controlling for muscle mass, nutritional biomarkers, MIS, fluid overload and RRF, showed that for every 1 mmol/l increase of sNa the odds of reduced HGS was decreased by 60% (OR = 0.40, 95% CI: 0.16–0.99) and 42% (OR = 0.58, 95% CI: 0.36–0.93) in HD and PD patients, respectively. However, stratified analysis indicated that lower sNa levels predicted reduced HGS in individuals with a background of malnutrition, inflammation, overhydration and less preserved RRF, representing unfavorable conditions strongly related to muscle wasting in the dialysis setting.

**Conclusions:** Predialysis sNa is a strong and independent determinant of HGS, a reliable nutritional marker in CKD-5D stage patients. However, according to our findings, lower sNa levels appear to be a marker of underlying unfavorable conditions that are heavily associated with reduced HGS, rather than a causal determinant of reduced HGS. Whether optimizing sNa levels improves patient muscle performance requires further investigations.

## Introduction

Protein-energy wasting (PEW) is defined as a pathological state where there is a continuous decrease or wasting of both protein deposits and energy reserves (1). PEW is highly prevalent among chronic kidney disease (CKD) patients (2) and is associated with high morbidity and mortality rates (3, 4). Handgrip strength (HGS) has been recommended by national guidelines (5) as a measure that can help to further assess nutritional state in those who are at risk of developing or have developed under-nutrition. HGS, a measurement of the maximal voluntary force of the hand/arm, has emerged as a simple and reliable method of assessing muscle function and indirectly the nutritional status in the general population (6) and patients with CKD (7, 8). Furthermore, several studies showed that reduced HGS could independently predict adverse health outcomes, such as inflammation, malnutrition, over-hydration, and higher mortality and morbidity in CKD populations (9–13). These data further reinforce the systematic use of HGS in the assessment of muscle function related to nutritional status in the CKD setting.

CKD is frequently complicated with hyponatremia (14, 15) which, according to recent studies, has emerged as a risk factor of mortality in both dialysis and non-dialysis dependent CKD patients (14–19). It remains, however, uncertain as to whether hyponatremia is a causal determinant of mortality in CKD patients or a marker of underlying conditions that are in and of themselves associated with death, such as malnutrition, inflammation, and fluid overload (17–19). Indeed, the predialysis serum sodium (sNa) was systematically associated with nutritional parameters including lean tissue index, body weight, and plasma creatinine and albumin concentrations. These data provide converging evidence that there are probably 2 main types of hyponatremic CKD patients: patients with poor nutrition and patients with disorders of sodium and fluid imbalance (18).

While both hyponatremia and reduced HGS are associated with poor nutritional status, data regarding the link between sNa and HGS are scarce. In a recent report (20) sNa was directly associated with HGS, but this association was lost after controlling for inflammation markers. This study aimed to (a) identify factors affecting HGS and predialysis sNa in CKD-5D patients, (b) explore the relationship between sNa and HGS, and (c) search for factors that could modify the association of sNa with HGS.

## Materials and Methods

### Study Population

The study population consisted of 73 CKD stage 5D patients (45 on hemodialysis and 28 on peritoneal dialysis) from the dialysis unit of the University Hospital of Heraklion, Greece. All of them had been on renal replacement treatment for at least 6 months before entering the study, were ambulatory, and aged between 18 and 90 years. Exclusion criteria included malignant disease, sepsis, concurrent inflammatory illness, inability to communicate effectively, and unwillingness to participate. Enrolled HD patients were on standard 4 h, 3 times weekly dialysis program, using bicarbonate dialysate and a dialysate Na of 138 mmol/L, whereas nine PD patients were on a standard continuous ambulatory PD program (CAPD) and nineteen on automated peritoneal dialysis (APD). Data about age, history of cardiovascular disease (CVD), and diabetes were retrieved from patients' medical charts. The study was approved by the institutional review board of the University Hospital of Heraklion and all subjects provided written consent before participation.

### Handgrip Strength

HGS was measured using a digital grip strength dynamometer (TKK 5401 GRIP D; Takei, Japan). Grip strength was measured with the elbow in full extension and the palm facing the body, while patients were seated upright with feet flat on the floor. HD patients were instructed to apply as much handgrip pressure as possible using the non-fistula hand, 20–40 min after dialysis session. When dialysis was complicated with intradialytic hypotensive episodes necessitating intravenous fluids infusion, all measurements assigned to that day (HGS, BIA, anthropometric parameters) were not performed and were post-poned to a later date. The maximal out of three repeating grip strength measurements with an interval of at least 5 s between was regarded as the handgrip strength. PD patients were asked to apply the maximum grip strength for 4 times, with alternating left and right hands. The average of the 4 measurements was regarded as the handgrip strength. According to the diagnostic criteria established by the European Working Group on Sarcopenia in Older People (EWGSOP), patients were diagnosed with reduced HGS, if HGS was below 30 and 20 Kg in men and women, respectively (21).

### Anthropometric Evaluation

Anthropometric measurements included body mass index (BMI), triceps skinfold thickness (TSF), mid-arm circumference (MAC), and mid-arm muscle circumference (MAMC). All measurements were taken by the same researcher, on the non-access site for HD patients, with the use of a Seca 703 digital scale (Seca Gmbh & Co.) and stadiometer (Seca 220), a set of Harpenden skinfold calipers (HSB-BI, British Indicators), and a common inextensible tape. Anthropometry took place either 20–40 min after the HD session or during an empty peritoneal cavity, for the PD patients. Each measurement was taken three times and the average value was recorded (22). MAMC was calculated according to the following formula: MAMC (cm) = MAC (cm) −3.14 × TSF (cm).

### Body Composition Analysis

Body composition analysis was carried out with Bioelectrical Impedance Analysis (BIA). The analysis was performed in a 50 kHz single-frequency system, using tetrapolar electrodes (BIA-101; RJL/Akern Systems, Clinton Township, MI, USA), according to the manufacturer's guidelines. The study was carried out 15–20 min following the HD session. Peritoneal dialysis patients emptied the peritoneal cavity before measurements. Average of two readings was obtained in all. Based on the bioelectrical measures of resistance (R) and reactance (Xc), obtained directly from the impedance signal, the following parameters were calculated, and used in data analysis: (a) fat mass (FM) and fat-free mass (FFM), which were standardized by squared height (m^2^) and expressed in kg/m^2^ as the fat mass index (FMI) and fat-free mass index (FFMI), respectively and (b) skeletal muscle mass (SMM), calculated by the following equation (23): SMM (kg) = ¼ * 0.566 * FFM which was standardized by squared height (m^2^) and expressed in kg/m^2^ as skeletal muscle index (SMI) and (c) extracellular water to total body water (ECW/TBW) ratio.

### Malnutrition-Inflammation Score

Malnutrition-inflammation score (MIS), as described by Kalantar-Zadeh et al. (24) was calculated for all patients. MIS consists of 4 domains, assessing patients' medical history, physical examination, BMI and laboratory parameters, and 10 components. The total score deriving from all MIS components ranges from 0 to 30, with higher scores reflecting an increased risk of malnutrition and inflammation.

### Laboratory Evaluation

For sNa values and all other laboratory measurements, the average of the last 6 months' values before the study was used. Blood samples were drawn from a peripheral vein before dialysis for HD patients and upon visit for PD patients. Serum levels of albumin (sAlb), glucose, creatinine, hemoglobin, total cholesterol, triglycerides, sodium, potassium, calcium, magnesium, phosphorus, and parathormone were determined using standard laboratory techniques.

Systolic (SBP) and diastolic blood pressures (DBP) were measured 30 min after the end of hemodialysis using an air manometer at the time of BIA investigation and are presented as the average of three consecutive measurements taken at 2-min intervals. Blood pressures were measured in PD patients with an empty abdomen using the same method. Residual renal function (RRF) was estimated as the mean renal clearance of urea and creatinine.

### Statistical Analysis

For all statistical analyses, the SPSS/PC 20 statistical package (Chicago, IL) was used. Given that dialysis treatment characteristics and mechanisms of hyponatremia differ by dialysis modality, separate analyses were performed for HD and PD patients. Normally distributed variables were expressed as mean ± SD and non-normally distributed variables were expressed as median (interquartile range). Chi-square test was used for the analysis of categorical variables and Student's *t*-test for continuous variables, as appropriate. Stepwise multiple logistic regression analysis was performed to determine significant factors associated with reduced HGS, according to the diagnostic criteria as mentioned above. Univariate and multivariate regression analyses were used to test the associations of variables related to handgrip strength and sNa. We also searched for variables that could modulate the relationship between sNa and reduced HGS. Thus, we conducted several stratified analyses in which categorization of the numerical variables was made according to their median. Statistical significance was set at the level of *P* < 0.05 (two-sided).

## Results

### General Characteristics

The study cohort consisted of 73 patients with a mean age of 61 ± 14 (range 21–89) years. Forty-five patients (27 men and 18 women, mean age 63 ± 14 years) were undergoing HD treatment and 28 patients (16 men and 12 women, mean age 57 ± 14 years) were treated with PD. Patients in the HD group as compared to those in the PD group had higher sAlb, serum potassium and MAMC, and lower RRF and residual urine volume (RUV) (Tables 1, 2). In addition, HD patients tended to be older in age (*p* = 0.074) with a longer dialysis vintage (*p* = 0.130), a lower predialysis sNa (*p* = 0.072) and less use of diuretics (*p* = 0.091).

**Table 1:** Characteristics of the patients on hemodialysis classified into normal and reduced handgrip strength (HGS).

**Characteristic**	**All patients *N =* 45**	**Normal HGS *N =* 15**	**Reduced HGS *N =* 30**	***P*^a^**	***r*^†^**	***P*^b^**
**Epidemiologic and clinical**						
Age (year)	63 ± 14	57 ± 13	64 ± 14	0.062	−0.250	0.098
Sex (male = 1/females = 2 (n)	60/40	11/4	16/14	0.197	−0.561	**0.000**
Diabetes mellitus (%)	22.2	20	23.3	0.800	−0.060	0.696
Cardiovascular disease (%)	48.9	40	53.3	0.399	−0.147	0.335
Dialysis vintage (months)	57 (12–112)	15 (8–63)	67 (17–134)	0.055	−0.390	**0.008**
Systolic blood pressure (mmHg)	135 ± 19	141 ± 16	132 ± 20	0.163	0.135	0.376
Diastolic blood pressure (mmHg)	72 ± 15	76 ± 14	70 ± 15	0.191	0.275	0.067
Residual renal function (ml/min)	0 (0–3)^*^	3 (0–6.75)	0 (0–0)	**0.012**	0.301	**0.044**
Residual urine volume (ml)	0 (0–1,500)^**^	200 (0–500)	0 (0–0)	0.071	0.263	0.081
Furosemide (%)	4.4	13.3	0	**0.042**	0.176	0.247
**Anthropometric**						
Body mass index (Kg/m^2^)	25.9 ± 4.2	25.7 ± 3.4	26.0 ± 4.6	0.799	0.009	0.953
Mid-arm circumference (cm)	30.2 ± 4.3	31.0 ± 3.8	29.8 ± 4.6	0.390	0.159	0.286
^*^Mid-arm muscle circumference (cm)	24.7 ± 3	25.7 ± 3.0	24.2 ± 2.9	0.128	0.331	**0.027**
Triceps skinfold thickness (cm)	1.72 ± 0.94	1.69 ± 1.01	1.74 ± 0.93	0.853	−0.087	0.572
**Bioimpedance**						
Fat-free mass index (Kg/m^2^)	17.4 ± 2.5	18.2 ± 2.2	17.0 ± 2.5	0.125	0.487	**0.001**
Fat mass index (Kg/m^2^)	8.5 ± 3.7	7.5 ± 3.0	9.0 ± 4.0	0.190	−0.316	**0.035**
Skeletal muscle index (Kg/m^2^)	8.4 ± 1.7	9.0 ± 1.4	8.1 ± 1.8	0.105	0.574	**0.000**
ECW/TBW ratio	46 ± 6	41 ± 4	48 ± 6	**0.001**	−0.414	**0.005**
**Nutritional and biochemical**						
Albumin (g/dl)	4.11 ± 0.26^*^	4.24 ± 0.22	4.05 ± 0.24	**0.012**	0.427	**0.003**
Glucose (mg/dl)	108 ± 39	98 ± 23	113 ± 45	0.210	−0.200	0.188
Creatinine (mg/dl)	8.5 ± 2.4	10 ± 1.9	7.7 ± 2.2	**0.001**	0.503	**0.001**
Hemoglobin (g/dl)	11 ± 1.2	11.1 ± 1.2	11.0 ± 1.2	0.877	0.101	0.509
Total Cholesterol (mg/dl)	161 ± 31	161 ± 35	160 ± 30	0.950	−0.117	0.443
Triglycerides (mg/dl)	138 (101–196)	152 (103–198)	129 (99–196)	0.097	0.164	0.280
Na (mmol/L)	138 (136–139)	139 (138–141)	137 (136–139)	**0.001**	0.462	**0.001**
K (mmol/L)	5.4 ± 0.7^**^	5.3 ±.7	5.4 ± 0.7	0.523	−0.058	0.703
Calcium (mg/dl)	9.0 ± 1.0	8.6 ± 1.5	9.2 ± 0.6	**0.039**	−0.209	0.167
Magnesium (mg/dl)	2.4 ± 0.3	2.4 ± 0.5	2.4 ± 0.2	0.355	−0.111	0.469
Phosphorus (mg/dl)	5.2 ± 1.6	5.3 ±.1.3	5.2 ± 1.7	0.842	0.091	0.551
Parathormone (pg/ml)	306 (178–617)	398 (175–1,022)	281 (177–517)	0.874	0.036	0.813
Malnutrition-inflammation score	4.6 ± 3.02	3.07 ± 2.52	5.37 ± 2.99	**0.014**	−0.426	**0.004**
Handgrip strength (Kg)	22.8 ± 10.1	33.5 ± 6.2	17.5 ± 6.9	**0.000**	–	–

*Values expressed as mean ± SD or median (interquartile range). Significant differences or correlations are shown with bold numbers. HD, hemodialysis; ECW/TBW, extracellular water/ total body water*.

P^a^ *P for normal HGS vs. reduced HGS*.

P^b^ *P for Pearson correlation coefficient*.

r^†^ *Pearson correlation coefficient between baseline characteristics and handgrip strength*.

* p < 0.05 vs. PD patients;

** *p < 0.001 vs. PD patients (shown in Table 2)*.

**Table 2:** Characteristics of the patients on peritoneal dialysis classified into normal and reduced handgrip strength (HGS).

**Characteristic**	**All patients *N =* 28**	**Normal HGS *N =* 13**	**Reduced HGS *N =* 15**	***P*^a^**	***r*^†^**	***P*^b^**
**Epidemiologic and clinical**						
Age (year)	57 ± 14	57 ± 13	64 ± 14	0.108	0.001	0.997
Sex (male = 1/females = 2 (n)	16/12	8/5	8/7	0.662	−0.682	**0.000**
Diabetes mellitus (%)	28.6	38.5	20	0.281	0.234	0.232
Cardiovascular disease (%)	46.4	38.5	53.3	0.431	−0.078	0.674
Dialysis vintage (months)	40 (32–67)	37 (14–46)	48 (33–72)	0.133	−0.228	0.253
Systolic blood pressure (mmHg)	131 ± 18	134 ± 13	127 ± 21	0.292	−0.026	0.897
Diastolic blood pressure (mmHg)	75 ± 15	74 ± 15	76 ± 16	0.690	−0.228	0.253
Icodexrein (%)	35.7	30.8	40	0.627	−0.171	0.384
CAPD = 1/APD = 2 (n)	9/19	5/8	4/11	0.523	−0.288	0.137
Residual renal function (ml/min)	2.33 (0–6.09)	1.5 (0–10.8)	2.37 (0–5.65)	0.341	0.289	0.136
Residual urine volume (ml)	550 (0–1,350)	300 (0–1,650)	700 (0–1,200)	0.889	0.116	0.555
Furosemide (%)	32.1	30.8	33.3	0.885	0.077	0.696
**Anthropometric**						
Body mass index (Kg/m^2^)	25.5 ± 4.2	26.3 ± 5.1	24.9 ± 3.2	0.372	0.169	0.390
Mid-arm circumference (cm)	29.4 ± 4.1	30.8 ± 4.9	28.2 ± 2.9	0.093	0.221	0.259
Mid-arm muscle circumference (cm)	23.1 ± 3.4	24.5 ± 3.0	21.8 ± 3.3	**0.032**	0.305	0.115
Triceps skinfold thickness (cm)	2.03 ± 0.9	2.05 ± 1.22	2.02 ± 0.54	0.947	−0.052	0.792
**Bioimpedance**						
Fat-free mass index (Kg/m^2^)	17.5 ± 2.5	18.8 ± 2.2	16.4 ± 2.2	**0.009**	0.567	**0.002**
Fat mass index (Kg/m^2^)	8.0 ± 3.9	7.6 ± 4.3	8.4 ± 3.6	0.596	−0.154	0.434
Skeletal muscle index (Kg/m^2^)	8.4 ± 1.6	9.2 ± 1.3	7.7 ± 1.6	**0.009**	0.689	**0.000**
ECW/TBW ratio	46 ± 5.3	47 ± 6	45 ± 5	0.591	−0.015	0.938
**Nutritional and biochemical**						
Albumin (g/dl)	3.98 ± 0.23	4.05 ± 0.08	3.92 ± 0.30	0.151	0.048	0.809
Glucose (mg/dl)	106 ± 54	93 ± 15	118 ± 72	0.220	−0.252	0.196
Creatinine (mg/dl)	9.0 ± 2.9	9.1 ± 3.4	8.9 ± 2.5	0.856	0.053	0.789
Hemoglobin (g/dl)	11.2 ± 1.5	10.9 ± 1.3	11.4 ± 1.7	0.422	0.101	0.509
Total Cholesterol (mg/dl)	165 ± 34	175 ± 45	155 ± 15	0.122	0.260	0.181
Triglycerides (mg/dl)	184 (152–192)	186 (150–229)	182 (150–187)	0.237	0.031	0.876
Na (mmol/L)	139 (137–141)	140 (139–143)	138 (136–140)	**0.008**	0.646	**0.000**
K (mmol/L)	4.6 ± 0.9	5.3 ±.7	5.4 ± 0.7	0.682	0.216	0.270
Calcium (mg/dl)	8.6 ± 1.2	8.5 ± 1.6	8.7 ± 0.9	0.711	0.206	0.293
Magnesium (mg/dl)	2.6 ± 0.6	2.6 ± 0.5	2.5 ± 0.6	0.601	0.065	0.742
Phosphorus (mg/dl)	4.9 ± 1.7	5.3 ±.1.8	4.6 ± 1.5	0.267	0.258	0.185
Parathormone (pg/ml)	335 (169–495)	322 (120–495)	436 (267–648)	0.078	0.045	0.820
Malnutrition–inflammation score	4.93 ± 2.19	4.69 ± 1.84	5.13 ± 2.50	0.605	−0.181	0.356
Handgrip strength (Kg)	24.5 ± 8.7	30.6 ± 6.2	19.3 ± 7.0	**0.000**	–	–

*Values expressed as mean ± SD or median (interquartile range). Significant differences or correlations are shown with bold numbers. PD, peritoneal dialysis; CAPD, continuous ambulatory peritoneal dialysis; APD, automated peritoneal dialysis; ECW/TBW, extracellular water/total body water*.

P^a^ *P for normal HGS vs. reduced HGS*.

P^b^ *P for Pearson correlation coefficient*.

r^†^ *Pearson correlation coefficient between baseline characteristics and handgrip strength*.

The baseline characteristics of the HD patients group are shown in Table 1. Patients with reduced, as compared to those with normal HGS, had lower sNa, sAlb, serum creatinine, RRF, and RRV(borderline) and higher MIS and ECW/TBW ratio. There were no other significant differences in patient characteristics between the two groups. The baseline characteristics of the PD patients group are shown in Table 2. Patients with reduced, as compared to those with normal HGS, had lower SMI, FFMI, MAMC, and sNa.

### Factors Associated With Reduced HGS

Factors associated with reduced HGS in the HD and PD patients groups were determined with univariate logistic regression analysis (Table 3). Controlling for factors with statistically significant association on univariate analysis (RRF, sAlb, MIS, and ECW/TBW ratio), multivariate logistic analysis in the HD group indicated that each increase in sNa by 1 mmol/l was associated with 60% (OR = 0.40, 95% CI: 0.16–0.99; *p* = 0.048) lower odds of having reduced HGS (Table 3). Also, sAlb and ECW/TBW ratio emerged as independent predictors of HGS in our multivariable model. The corresponding analysis in the PD group, after controlling SMI and MAMC, showed that each increase in sNa by 1 mmol/l was associated with 42% (OR = 0.58, 95% CI: 0.36–0.93; *p* = 0.026) lower odds of having reduced HGS (Table 3).

**Table 3:** Multiple logistic regression analysis to predict risk factors for developing reduced handgrip strength in 45 HD (A) and 28 PD patients (B).

	**Parameter**	**Univariate**		**Multivariable**	
		**OR (95%CI)**	***P***	**OR (95%CI)**	***P***
A	RRF (↑1 ml/min)	0.72 (0.55–0.96)	0.023	–	–
	MIS (↑ 1 point)	1.4 (1.05–1.88)	0.023	–	–
	sAlb (↑ 1 mg/dl)	0.3 (0.00–0.56)	0.019	0.002 (0.00–0.61)	0.031
	Na (↑ 1 mmol/L)	0.46 (0.26–0.77)	0.004	0.40 (0.16–0.95)	0.048
	ECW/TBW ratio(↑1 %)	1.28 (1.08–1.52)	0.005	1.32 (1.0–1.75)	0.049
B	SMI (↑Kg/m^2^)	0.47 (0.25–0.89	0.020	–	–
	MAMC (↑1 cm)	0.76 (0.58–0.99)	0.044	0.71 (0.51–1.00)-	0.049
	Na (↑1 mmol/L)	0.62 (0.41–93)	0.021	0.58 (0.36–0.93)	0.026

*RRF, residual renal function; MIS, malnutrition-inflammation score; sAlb, serum albumin; ECW/TBW, extracellular water/total body water; SMI, skeletal mass index; MAMC, mid-arm muscle circumference*.

### Determinants of HGS

In the HD group, HGS was positively associated with male gender, RRF, MAMC, FFMI, SMI, sAlb, creatinine, and sNa, whereas it was inversely associated with HD vintage FMI, ECW/TBW ratio and MIS (Table 1). In a forward stepwise multiple regression analysis, where variables (sex, ECW/TBW ratio, MIS, SMI, sAlb) significant in univariate analysis were included, SMI, MIS, sAlb, and predialysis sNa emerged as significant independent determinants of HGS (Table 4). The results remained identical when sex and ECW/TBW ratio were substituted for RRF and dialysis vintage in the regression model. Together, these four variables explained 54% of the variance in HGS, whereas the contribution of sNa alone to the variance of HGS was ~4%. The corresponding multiple regression analysis in the PD group showed that sNa along with SMI and male gender explained 67% of the variance in HGS, whereas the contribution of sNa alone to the variance of HGS was ~6%.

**Table 4:** Multiple regression analysis of factors that affect handgrip strength (HGS) in 45 HD patients (A) and 28 PD patients (B) and subgroup analysis stratified by SMI categories: Low SMI (LSMI) and high SMI (HSMI) in the whole group (73 patients).

**A**	**Parameter**	**B**	**Standard error**	**Standard beta**	***P***	**Partial *r***	**Adjusted *R*^**2**^ change**
	SMI	2.494	0.629	0.427	0.000	0.531	0.31
	MIS	−0.930	0.367	−0.277	0.018	−0.372	0.45
	sAlb	9.759	4.206	0.247	0.026	0.344	0.50
	sNa	1.162	0.528	0.246	0.034	0.329	0.54
B	SMI	1.539	0.789	0.291	0.063	0.370	0.45
	Gender (female)	−7.591	2.184	−0.440	0.002	−0.579	0.61
	sNa	1.129	0.501	0.316	0.034	0.418	0.67
LSMI	sNa	1.744	0.57	0.448	0.004	0.47	0.31
	SMI	2.743	1.279	0.314	0.039	0.35	0.38
HSMI	Gender (female)	−10.597	2.962	−0.501	0.001	−0.53	0.20
	sNa	1.183	0.443	0.374	0.012	0.42	0.32

*Standard beta, standardized regression coefficients; r, correlation coefficient; SMI, skeletal mass index; MIS, malnutrition-inflammation score; sAlb, serum albumin*.

Next, to further explore the interrelationships among muscle mass (SMI), muscle function (HGS) and sNA, we included the interaction term SMI*sNa in both the above described regression analyses and repeated them. In both analyses the interaction term was statistically significant (*p* < 0.001). Then we examined potential predictors of HGS in the whole study population (73 patients), using SMI as dichotomous variable (dichotomized into high/low SMI using dialysis mode-specific SMI median cutoffs). Medians of SMI in HD and PD groups were 8.6 and 8.5 kg/m^2^, respectively. Among patients with low SMI, forward stepwise multiple regression analysis showed that sNa after controlling for univariately significant variables (i.e., SMI, MIS, HD vintage, and sex), emerged along with SMI as the only independent predictors of HGS (Table 4). In the corresponding analysis in the high SMI group, Na and sex emerged as the only significant predictors of HGS (Table 4).

In this context, the existed strong correlation (*r* = 498; *p* = 0.002) between HGS and SMI in the low SMI group was vanished in the high SMI group.

Interestingly, sNa showed a high diagnostic power for reduced HGS cases in the low SMI but not in the high SMI group. In the former group, the area under the ROC curve (AUC)for predicting the development of reduced HGS was 0.89 [(0.77–1.00); *p* = 0.001] and at an optimal sNa cutoff of 138 mmol/L the sensitivity and specificity were 79 and 78%, respectively. The corresponding AUC 0.684 [(0.51–0.86; *p* = 0.059)] in the high SMI group differed significantly (*p* = 0.046) from that in the low SMI group. These finding indicate: (a) that muscle strength is different from muscle mass, since in well-nourished patients (high SMI group) muscle mass and muscle strength are not directly correlated to each other, (b) the contribution of sNa to HGS variance was more than 2 1/2-fold greater in the low SMI than in high SMI group (31 vs. 12%), implying that the sNa has a differential impact on HGS in different SMI (proxy of nutritional status) strata and (c) the high ability of sNa to predict reduced HGS in malnourished patients is significantly reduced in well-nourished patients.

### Predictors of Predialysis sNa

As shown in Table 5, RRF and serum phosphorus were positive predictors, whereas ECW/TBW ratio, MIS, HD vintage and serum glucose were negative predictors of sNa in the HD group. In the PD group, SMI, FFMI, RRF, sAlb, and serum K were identified as positive predictors, whereas icodextrin was identified as a negative predictor of sNa.

**Table 5:** Indicators correlated with predialysis sNa in 45 HD patients (A) and 28 PD patients (B).

	**Indicator**	***r***	***P***
A	Residual renal function	0.249	0.046
	ECW/TBW ratio	−0.417	0.04
	MIS	−0.369	0.013
	Dialysis vintage	−0.300	0.046
	Serum Glucose	−0.297	0.028
	Serum phosphorous	0.299	0.046
B	Skeletal muscle index	0.601	0.001
	Fat-free mass index	0.513	0.05
	Residual renal function	0.400	0.035
	Icodextrin use	−0.508	0.006
	Serum albumin	0.425	0.024
	Serum potassium	0.398	0.036

*r, Pearson correlation coefficient; ECW/TBW, extracellular water/total body water; MIS, malnutrition-inflammation score*.

### Analysis of Factors Modifying the Association Between Predialysis sNa Levels and HGS

Stratification analyses after significant interaction terms indicated that potential effect modifiers of the sNa-HGS association were MIS, ECW/TBW ratio, sAlb, and RRF in the HD group. The corresponding effect modifiers in the PD group were sAlb and TSF (Table 6). Thus, the association between lower sNa levels and reduced HGS was apparent only in those HD patients with higher levels of MIS and ECW/TBW ratio, lower levels of sAlb and loss of RRF. Likewise, the association existed only for PD patients with lower levels of sAlb and TSF.

**Table 6:** Association between predialysis sNa levels and reduced handgrip strength after stratification of different study variables in 45 HD patients (A) and 28 PD patients (B), adjusted.

	**Parameter**	**OR (95%CI)**	***P***	**P for interaction**
A	MIS ≤ 4	0.36 (0.16–0.78)	0.365	0.024
	MIS > 4	0.68 (0.30–1.58)	0.010	
	ECW/TBW ratio ≤ 45	0.26 (0.08–0.88)	0.417	0.034
	ECW/TBW ratio > 45	0.76 (0.37–1.72)	0.031	
	sAlb ≤ 4.1 mg/dl	0.28 (0.08–0.98)	0.048	0.016
	sAlb > 4.1 mg/dl	0.43 (0.16–1.14	0.090	
	RRF ≤ 0	0.52 (0.27–0.99	0.046	0.030
	RRF > 0	0.45 (0.14–1.49)	0.193	
B	sAlb ≤ 4 mg/dl	0.49 (0.28–0.94)	0.030	0.051
	sAlb > 4 mg/dl	0.76 (0.43–1.33)	0.332	
	TSF ≤ 2	0.62 (0.39 −0.99)	0.045	0.017
	TSF > 2	0.70 (0.27–1.80)	0.464	

*MIS, malnutrition-inflammation score; ECW/TBW, extracellular water/total body water; sAlb, serum albumin; RRF, residual renal function; TSF, triceps skinfold thickness*.

## Discussion

The present study showed that lower predialysis sNa concentrations were associated with a greater risk of reduced HGS. This association remained statistically significant upon adjustment for muscle mass, malnutrition, inflammation, fluid overload and RRF, factors that might confound the observed association. We showed that for every 1 mmol/l increase of sNa the odds of reduced HGS, as defined in Methods, was decreased by 60 and 42% in HD and PD patients, respectively. Equally notable, there was an average 1.16 and 1.12 Kg increase in HGS for every 1 mmol/L increase in sNa in HD and PD patients, respectively. Furthermore, the results of our stratified analysis indicated that the association between lower sNa levels and reduced HGS was most apparent in malnourished HD patients with higher levels of inflammation, higher degrees of over-hydration, lower levels of RRF and in hypo-albuminemic PD patients with anthropometric evidence of under-nutrition by TSF.

In this study, we confirm many of the associations between HGS and variables used in the assessment of muscle mass and nutritional status in CKD stage 5D patients (9). Specifically, the negative correlations of HGS with age, MIS, and ECW/TBW and the positive ones with male gender, MAMC, FFMI, SMI, sAlb, creatinine, and RRF are in agreement with previous studies (7–9) and support the validity of our dataset. More importantly, this study documents for the first time the existence of a strong positive association between predialysis sNa and HGS in HD and PD patients, independently of the presence or absence of malnutrition, inflammation, fluid overload and RRF. In addition, the observed dissociation of muscle strength (HGS) and mass (SMI) in malnourished patients, but not in the well-nourished patients, as it was detailed in RESULTS, are findings to further support the notion that muscle strength and muscle mass are not always congruent and these two entities may be affected by different risk factors (25). In the study by Isoyama et al. (25), muscle strength showed a stronger association with PEW, inflammation and mortality than muscle mass, whereas other authors (26) suggest that low muscle mass may be secondary to the effects of low muscle strength.

We also explored the predictors of sNa separately in HD and PD patients (Table 5). In the HD group, sNa was positively correlated with RRF and serum phosphorous and inversely correlated with MIS (a proxy of malnutrition-inflammation), ECW/TBW ratio (a proxy of fluid overload), HD vintage and blood glucose levels, whereas in PD group, sNa was positively correlated with SMI, FFMI, sAlb, phosphate, and potassium (proxies for nutritional status) and RRF, whereas the use of icodextrin was a significant negative predictor of sNa. These results agree with previously published literature. Indeed, in many epidemiological studies, sNa was associated with various skeletal muscle mass indices and nutritional-related parameters in both HD (15, 16, 18, 19, 27) and PD patients (16, 28, 29). Inflammation (a raised CRP > 6 mg/l), has been found (19) as an important predictor of hyponatremia and also MIS is inversely correlated with sNa level (30). Dysnatremia has been long recognized as a potential consequence of fluid overload and increased interdialytic weight gain (10, 27, 31–33). RRF has been identified as a key determinant of sNa in HD (16, 27) and PD populations (17, 28, 29), suggesting that RRF even in the advanced stage can contribute to sodium handling and prevent hyponatremia by excreting relatively more water than sodium. The observed inverse association between sNa and hyperglycemia in dialysis patients due to water shifts from the intracellular to the extracellular compartment is well-documented (15, 18, 27, 29, 34). Similarly, the reported association of icodextrin with lower sNa levels (28, 35, 36) is likely related to a dilutional effect due to an osmotic gradient caused by blood levels of icodextrin and metabolites. A very comprehensive review of hyponatremia in the dialysis population has been recently published (37).

It remains uncertain as to whether the aforementioned HGS-sNa association in the dialysis setting is causal, or due to confounding by underlying conditions that predispose to dysnatremia. In the present study we used stratified analysis to search for specific levels of confounders, in which low sNa levels could present a differentiated association with reduced HGS. Our results showed that in the HD cohort, the association was evident in overhydrated patients, with loss of RRF, manifesting signs of malnutrition and wasting, whereas the corresponding association existed in PD patients with low levels of albumin and TSF. These data show that sNa is a marker of underlying unfavorable clinical conditions that are heavily associated with reduced HGS. Thus, interventions for increasing dietary protein and energy intake, reducing inflammation, optimizing fluid status and preserving RRF could eliminate underlying causes of low sNa levels and consequently ameliorate the risk of reduced HGS. In this context, the pre-dialysis sNa concentration appears to be unaffected by the dialysate sodium concentration (16, 38) and thus, efforts to favorably impact predialysis sNa levels by using personalized dialysate sodium prescription or different fixed dialysate sodium concentrations may not have the expected effect.

Despite the presence of the effect modifiers identified by our stratified analyses, low levels of sNa, even in the normal range, remained an independent risk factor for reduced HGS, after extensive adjustment of confounders, findings indicative of a possible direct effect of sNa on muscle function. Such an effect might be plausible since sodium concentrations affect the 3-dimensional conformations of proteins and enzymes and play a critical role in nerve-impulse transmission, muscle excitation, and maintenance of transmembrane-electrical gradients that are critical to cellular function. In this context, a recent metanalysis showed that hyponatremia was associated with a significantly increased risk of falls (39, 40). The risk of falls can be explained by the impact of hyponatremia on muscle strength as it was addressed in animal studies (41) and peripheral nerve system in humans (42).

Irrespective of the pathogenetic link between HGS and sNa, several of our findings deserve further mention. First, in agreement with previous reports, this study identified several shared risk factors for lower sNa levels across the two dialysis modalities, including poor nutritional status (15, 16, 28) and loss of RRF (19, 29). Second, the fact that lower sNa levels in both HD and PD patients groups were highly predictive of impairments in muscle function, came at no surprise, since in several studies in dialysis patients, lower sNa levels, even in the normal range, have been associated with adverse outcomes, including a higher risk of all-cause mortality (15, 16, 19). Third, sodium intake *per se* is closely correlated with protein, energy carbohydrate and potassium intake in both HD (43) and PD populations (44), showing that sodium is inherent in much of the food commonly consumed, and therefore closely associated with the amount of food consumed. It is worth mentioning that low dietary sodium intake has been associated with adverse outcomes in dialysis patients (44) and the general population (45), independently of protein and energy intake. In the present study, we did not assess intakes from sodium and other nutrients, and therefore we cannot further comment on the way sodium intake could influence sNa levels and HGS. At this point it is worth noting that the benefit of fluid restriction to avoid hyponatremia is achieved only if optimal nutritional status and food intake is not compromised. Large interdialytic weight gain and/or fluid overload is not a uniform phenomenon and can represent two opposite ends of the spectrum of inflammation malnutrition syndrome: cachectic inflamed subjects with associated hyponatremia on one side and well-nourished patients with high-salt intake between dialysis treatments patients. This heterogeneity associated with large interdialytic weight gain explains the discrepancy between studies examining the relationship between IDWG and nutritional status. Pre-dialysis serum sodium can help differentiate between these two different groups of patients and identify the subgroup of patients where dietary changes are more likely to be effective. Forth, our observations that the nutritional biomarkers serum albumin and potassium and the anthropometric measurement MAMC were lower in the PD than in HD group, indicate that HD patients remain in a better nutritional status than PD patients. However, the tendency toward a higher HGS in PD than in HD patients (24.5 ± 8.7 vs. 22.8 ± 10.1 Kg) somewhat contradicts the result of worse nutrient intake in the PD patients, that can be explained by the fact that PD patients were younger with better preserved RRF and RUV than HD patients. Also, the strong association between sNa and RRF in both HD and PD patients helps to explain the lower sNa in HD than in PD patients. In this regard, a recent study (46) assessed for a first time the nutrient intake by the Semi-FFQ and dietary behavior according to dialysis modality, and showed that nutritional status (serum albumin and potassium), dietary behaviors, and nutrient intake-to-recommended allowance ratio were worse in PD patients compared to HD patients. Despite this, HGS was significantly higher in PD patients than in HD patients, which was attributed to the lower age of PD. All these data taken together, suggest that reduced HGS has a multifactorial etiology, and multiple causes of reduced HGS may be identified in individual patients, requiring, thus, an individualized therapeutic approach.

At this point, it should also be emphasized that physical activity interventions are warranted in patients with CKD of all stages and treatments, regardless of age, because their lower levels of physical activity and physical functioning, compared to those of general population of elderly adults, are associated with poor clinical outcomes (47, 48). Exercise training in these patients can increase exercise capacity, improve muscle strength and function, decrease blood pressure, and improve inflammation and oxidative stress biomarkers (49). HGS is linearly related to daily physical activity in older men and women (50) and intradialytic resistance exercise, a form of exercise that improves muscular strength and endurance, has been shown to effectively improve physical activity (HGS) and attenuate inflammatory responses, which might contribute to the beneficial effects of exercise (51).

This study may have also clinical implications. Since this was a hypothesis-generating study, further study to prove a causal relation between HGS and sNa is mandatory. This can be achieved in studies, after adjustment for baseline covariates, by altering sNa by any means (manipulating dialysate Na concentration, Na intake) and examining the direct effects (short-term) on HGS. If such a causal link is identified, then the application of this knowledge may be of help in dialysis patients with low sNa, but more specifically in fluid overloaded malnourished dialysis patients, where HGS was substantially dependent on sNa. In these patients, preventing or treating malnutrition that relate to low sNa may ameliorate the risk of reduced HGS. However, this is not an easy task; it may take a long time to be achieved and sometimes is unachievable. Increasing sNa on its own may improve at least muscle strength and consequently clinical outcomes. New diagnostic and sodium balancing tools (modern biosensors combined with advanced analytics embedded in the dialysis machine and even novel technologies to quantitate tissue salt accumulation) that allow for a more personalized therapeutic interventions on sodium and water management (52) may help mediating this HGS improving effect of sNa on malnourished dialysis patients. The study has several limitations. First, the sample size of the study was small and, therefore, the predictability of sNa for HGS and nutritional outcomes should be prospectively verified in larger studies. Second, we were unable to account for the dietary amount of sodium being ingested in the diet or removed by the dialysis procedure and the overall fluid balance such as dietary water intake, ultrafiltration rates and body weight changes, which may have resulted in residual confounding. Third, we acknowledge that there were baseline imbalances in demographic and clinical variables in patients with normal and reduced HGS, such as age, HD vintage, residual GFR and several other nutritional parameters, causing thus confounding; however, great efforts were made to adjust for these baseline confounders by extensively using multivariable regression analysis and stratification, methods most frequently used to control for confounding. By doing so, we restricted conditional bias from covariate imbalance. In this regard, sNa was accountable for 4 and 6% of the variation of HGS in the HD and PD groups, respectively, whereas after stratified analysis in the whole group, it was accountable for 31 and 12% in the malnourished (low SMI) and well-nourished (high SMI), respectively. Fourth, although the robustness of the HGS-sNa association did not decrease even after extensive adjusting for demographic, anthropometric, bioimpedance, clinical and laboratory covariates, causality cannot be established in the setting of an observational study. Fifth, a strength of this study is that the sNa -HGS association was detected in both HD and PD patients, who differ in many important ways, including regulation of sodium and water balances, RRF status, and potentially the diet patterns and nutritional intake (46), to name some of them.

## Conclusions

We showed that predialysis sNa levels impact substantially on muscle mass and function, as measured by SMI and HGS, respectively, emerging thus as an important predictor of muscle wasting in the context of CKD stage 5D. However, lower sNa levels predicted reduced HGS in subsets of patients with a background of malnutrition, inflammation, abnormalities in fluid status and less preserved RRF, representing unfavorable conditions strongly related to muscle wasting, one of the best markers of PEW, in the dialysis setting. These findings suggest that lower sNa levels perform as a marker of the overall risk of HGS reduction rather than as a direct pathogenic factor. Whether optimizing sNa levels improves muscle strength in CKD 5D stage requires further investigations.

## Data Availability Statement

The raw data supporting the conclusions of this article will be made available by the authors, without undue reservation.

## Ethics Statement

The studies involving human participants were reviewed and approved by Heraklion University Hospital Ethics Committee. The patients/participants provided their written informed consent to participate in this study.

## Author Contributions

AM, PK, and E-KD wrote the article. SM, EP, AS, and HD made all anthropometric measurements. DL and MG made the body composition analysis. IP made the statistical analysis. KS interpreted the data, corrected the manuscript, and submitted the final version. All the authors read and approved the final manuscript.

## Conflict of Interest

The authors declare that the research was conducted in the absence of any commercial or financial relationships that could be construed as a potential conflict of interest.
